# Resource consumption of multi-substance users in the emergency room: A neglected patient group

**DOI:** 10.1371/journal.pone.0223118

**Published:** 2019-09-26

**Authors:** Laurence Klenk, Christina von Rütte, Jonathan F. Henssler, Thomas C. Sauter, Wolf E. Hautz, Aristomenis K. Exadaktylos, Martin Müller

**Affiliations:** 1 Department of Emergency Medicine, Inselspital, Bern University Hospital, University of Bern, Bern, Switzerland; 2 Department of Psychiatry and Psychotherapy, St. Hedwig Hospital Berlin, Charité University Medicine, Berlin, Germany; 3 Medical Skills Lab, Charité Medical School Berlin, Berlin, Germany; 4 Institute of Health Economics and Clinical Epidemiology, Cologne University Hospital, Cologne, Germany; University of Genoa, ITALY

## Abstract

**Background:**

Multi-substance use is accompanied by increased morbidity and mortality and responsible for a large number of emergency department (ED) consultations. To improve the treatment for this vulnerable group of patients, it is important to quantify and break down in detail the ED resources used during the ED treatment of multi-substance users.

**Methods:**

This retrospective single centre case-control study included all ED consultations of multi-substance users over a three-year study period at a university hospital in Switzerland. Resource consumption of these patients was compared to an age-matched control group of non-multi-substance users.

**Results:**

The analysis includes 867 ED consultations of multi-substance users compared to 4,335 age-matched controls (5:1). Multi-substance users needed more total resources (median tax points [medical currency] (IQR): 762 (459–1226) vs. 462 (196–833), p<0.001), especially physician, radiology, and laboratory resources. This difference persisted in multivariable analysis (geometric mean ratio (GMR) 1.2, 95% CI: 1.1–1.3, p = 0.001) adjusted for sociodemographic parameters, consultation characteristics, and patient comorbidity; the GMR was highest in ED laboratory and radiology resource consumption. Among multi-substance user, indirect and non-drug-related consultations had higher ED resource consumption compared to drug-related consultations. Furthermore, leading discipline as well as urgency were predictors of ED resource consumption. Moreover, multi-substance users had more revisits (55.2% vs. 24.9%, p<0.001) as well as longer ED and in-hospital stays (both: GMR 1.2, 95% CI: 1.1–1.3, p<0.001).

**Conclusion:**

ED consultations of multi-substance users are expensive and resource intensive. Multi-substance users visited the ED more often and stayed longer at the ED and in-hospital. The findings of our study underline the importance of this patient group. Additional efforts should be made to improve their ED care. Special interventions should target this patient group in order to decrease the high frequency and costs of emergency consultations caused by multi-substance users.

## Introduction

Substance abuse in Western countries is increasing [1]. It causes more than a quarter of a billion disability-adjusted life years worldwide–particularly in Europe [2] and is associated with a high mortality and morbidity [3,4].

Multi-substance users frequently present to emergency departments (ED) [3,5–10] and nearly half of ED visits in the US are associated with substance abuse [11]. In addition, multi-substance use is often associated with significant comorbidities both mental health and medical disorders [7]. Consequently, multi-substance users may require substantial ED resources and have an increased risk of revisit after discharge, thus further increasing resource consumption and ED load [12–14].

We believe that efficient treatment of this vulnerable patient group not only affects ED resources but on the same hand might reduce side effects and discomfort originating from potentially unnecessary examinations. However, whether this widely suspected resource need really exists and where in the process of ED care it occurs is poorly understood as research on this topic is sparse. Yet, such knowledge is required in order to develop targeted interventions with the highest potential to be effective and efficient. Therefore, the aim of this study was i) to compare ED resource consumption of multi-substance users with an age-matched control group of non-multi-substance users, ii) to identify where in the ED process the largest differences in resource consumption originate, and iii) which parameters among multi-substance users predict the ED resource consumption.

## Methods

### Study design and setting

This is a retrospective single centre case-control study. The study site was the adult ED (level 1) of the university hospital of Bern, the capital of Switzerland. A mean of more than 35,000 patients per year were treated over the study period.

### Definition of multi-substance user

In light of the complex and inconsistent use of terminology of multi-substance abuse [4] we used a pragmatic approach, defining multi-substance use as:

- any abuse of, or addiction to, two or more psychotropic substances, according to WHO ICD-10 criteria of abuse and addiction (ICD-10: F10-F19), including indiscriminate abuse of polyvalent substances (ICD-10: F19).

To ensure clinical relevance, patients with simultaneous alcohol- and tobacco abuse only–without concurrent abuse/addiction of any other psychotropic substances–were not included in this study. This pragmatic approach was agreed upon after consultation with the Swiss Federal Office of Public Health.

### Eligibility criteria

All consultations of adult patients (≥16 years) were eligible for this study when there was a documented diagnosis of multi-substance use on admission to the ED during the three-year study period between May 11, 2012 and May 10, 2015.

Pregnant women usually present to the separate gynaecology and obstetrics department. Younger patients (<16 years) are routinely treated in the paediatric ED and are therefore not included in our study population. Consultations of patients with a previous–but no longer active–diagnosis of multi-substance use were excluded, as were consultations of patients who had refused to provide general consent for the anonymous use of their patient data, and consultations with insufficient information on resource consumption. Furthermore, consultations with incomplete information on the potential confounding variables (see below) and consultations in which the primary attending discipline was psychiatry (no comprehensible resource expense documentation) were excluded.

### Primary and secondary outcomes

The primary outcome was the total ED resource consumption defined as the sum of physician, nurse, laboratory, and radiology resources (see below). Resources were measured in tax points (TP, medical currency). TP are a measure constructed for billing purposes. For single services (e.g. patient time), the tax points correspond to minutes of work (TARMED Suisse, TARMED, 01.06.2012). One TP is about 0.89 CHF, but the exact value depends–among others–on the hospital size. In contrast to the total ED costs, the sum of the billed TP reflects the resource needs of a patient.

The following secondary outcomes were analysed:

- Resource subgroups: physician resources divided in total resources [TP] as well as patient, admin, and medical report time [minutes], total nurse resources, laboratory resources, and radiology resources [TP]; in addition to the total radiology resources, the binary outcome parameter sonography, X-ray, computer tomography (CT) scan, and magnetic resonance tomography (MRT) scan performed [yes/no] were evaluated.
- Administrative outcomes: the total ED costs [Swiss Francs], length of ED stay [minutes] and length of hospital stay [days].
- Clinical outcomes: intensive care unit (ICU) admission and in-hospital mortality.

### Potential confounder/resource predictors

A multivariable statistical model was adjusted for the following covariables:

- Sociodemographic parameters: age, sex, and insurance type (general vs. private),
- Consultation acuity: triage category, which is routinely registered by special qualified nurses with the Swiss Emergency triage scale (1 being highest, 5 lowest urgency), resuscitation room use [15], and type of admission (walk-in, involuntary admission, previous medical contact, ambulance, other).
- Consultation characteristics: night admission (19:00–06:59), weekend admission (Saturday/Sunday), season (spring, summer, fall, winter), revisit, and area of primary problem (surgical, internal, or others such as otorhinolaryngology and ophthalmology).
- Patient comorbidity: the Charlson comorbidity index [16], including comorbidities such as chronic kidney disease, liver disease, chronic obstructive pulmonary disease (COPD), cardiovascular disease, dementia, cancer, and diabetes mellitus summed up to a comorbidity index, was determined.

To further predict resource consumption between multi-substance users, the following multi-substance user specific parameters were determined: i) drug-related consultation grouped in directly related such as intoxications and abscesses after drug injection, indirectly such as Hepatitis complication after drug-use, and non-related, ii) documented intravenous drug-abuse as well as iii) heavy-ED user consultation defined as more than 3 consultations in one year [5].

### Data extraction

Potentially eligible consultations were identified through a full text keyword search (“polytoxicomania” with different semantic combinations) in the comprehensive emergency medical report. The medical report contains patient history, admission procedure as well as established and chronic diagnoses. The ED medical report is routinely electronically generated and stored by the physician in charge in our computerised patient database (E-Care, ED 2.1.3.0, Turnhout, Belgium).

The complete, anonymised medical report–including controller data on resource consumption (see below)–was exported to Microsoft Excel (Microsoft Corporation, USA) for further analysis. The medical report was analysed in full text by one study investigator (CR) to check for an active diagnosis of multi-substance use and ensure a fulfilling of the eligibility criteria and presented definition.

In addition to the potential confounder mentioned above, the clinical outcome parameters, ICU admission and in-hospital mortality, were extracted from our computerised patient database. The acute diagnoses were classified manually as medical, surgical, or psychiatric diagnosis, as shown in S1 Table. Furthermore, the reports of the multi-substance users were screened in full-text, to determine if intravenous drug consumption was documented and the diagnosis and history field was used to group the consultations in direct, indirect or no drug-related.

Staff members routinely document their work for each patient in the medical database for billing. Regular trainings for every staff member are performed to ensure a valid billing. Variables describing physicians’ work (total resources, patient time, administrative time, report time), nurses’ work, radiology diagnostics (total resources as well as X-ray, ultrasound, CT scan, and MRT), laboratory resources, and total costs (CHF, Swiss francs) were used to analyse resource consumption. All resource variables were measured in TP.

### Statistics

For further statistical analysis, the data was imported into Stata® 13.1 (StataCorp, The College Station, Texas, USA).

To each consultation, five randomly selected, age-matched consultations with complete data on the potential confounder variables were matched.

As all continuous variables were not normally distributed (Shapiro-Wilk test), the continuous variables were presented as medians with 25^th^ - 75^th^ interquartile ranges (IQR). Categorical variables were shown as per cent, accompanied by the absolute number. All continuous variables were ln-transformed to take into account the non-normal distribution of the parameter.

Univariable associations between being a multi-substance user (case vs. control) and categorical variables were tested using a chi square test. Differences in continuous variables between cases and controls were analysed using the Wilcoxon rank sum test.

Multivariable regression analysis on the ln-transformed continuous outcomes or logistic regression analysis for the binary outcomes adjusted for the potential confounder were used to quantify the association between being a multi-substance user and the outcomes. While the measure of strength in the logistic regression was odds ratio (OR), the measure of strength in linear regression with ln-transformed outcome was the geometric mean ratio (GMR) of the original outcome (exponentiated coefficients). Both measures are presented with their 95% Confidence Interval (CI).

A multivariable linear regression analysis was also used, to identify total ED resource predictors among multi-substance user. To obtain a final model in the linear regression models, parameters with p>0.2 were stepwise removed from the models.

For sensitivity analysis, the final model was varied using a conditional linear regression analysis (matched analysis). As subgroup analysis, we restricted the main analysis to the first consultation of an included multi-substance user to take into account that follow-up visits of a multi-substance user might not be independent events.

A p-value of less than <0.005 was considered significant to reduce false positive results [17].

## Results

**Fig 1:**
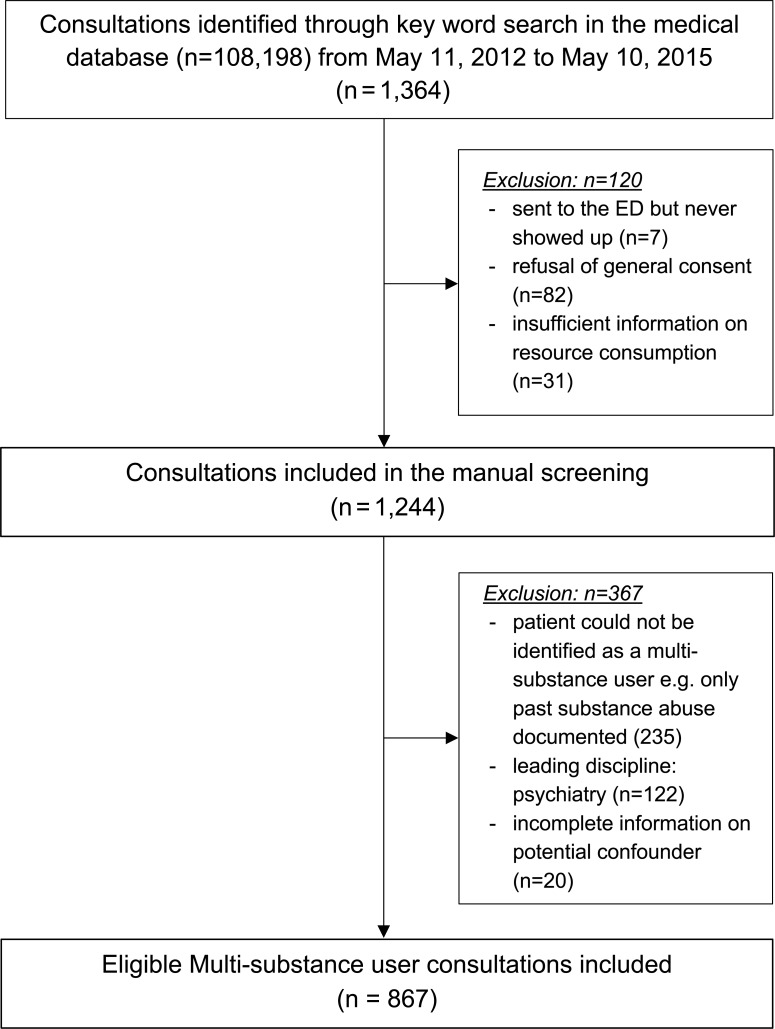
Flowchart of the study.

Our search resulted in the identification of 1,364 potentially eligible consultations of multi-substance users during the three-year study period (May 11, 2012 and May 10, 2015). In total, 8.8% (n = 120) of these had to be excluded because i) the patient was sent to the ED by another health care professional but never showed up (n = 7), ii) the patient had denied the general consent for the detailed use of anonymised patient data in clinical trials (n = 82), or iii) there was insufficient information on resource consumption (n = 31); see Fig 1. In manual screening (CR), 235 patients did not have an active substance use (e.g. only past substance use documented). Thus, 1,009 consultations out of 108,198 ED consultations were identified as multi-substance users leading to a proportional incidence of an ED consultation by a multi-substance user of 0.93%. Of the identified 1,009 multi-substance users, 142 had to be excluded for the final analysis: 122 consultations were solely seen and treated by a psychiatrist without comprehensible resource expense documentation and in further 20 consultations, information on the potential confounders was incomplete.

Consequently, 867 non-psychiatric ED consultations of documented multi-substance users were included in the detailed analysis. To each consultation, five randomly selected, age-matched consultations out of the 106,834 ED consultations with a negative result in key-word search with complete data on the confounder variables were matched. Therefore, the control group consisted out of 4,335 consultations.

### Baseline characteristics

The median age of the multi-substance user was 43 (IQR 34–49) years. Compared to the age-matched control group, the following significant differences were found (all p<0.001, Table 1): In regard to sociodemographic parameter, multi-substance users were more often males (66.9% vs. 58.3%) and less often private insured (0.2% vs. 8.8%). The consultation characteristic was more urgent (high urgent: 29.6% vs. 20.7%) and internal medicine visits (67.4% vs. 35.4%) as well as revisits (55.2% vs. 24.9%) were more often, walk-in was less often found (32.5% vs. 69.7%) in multi-substance users. No significant differences were found in night (p = 0.733) and weekend admissions (p = 0.115), over the different seasons of the year (p = 0.234) as well as in resuscitation room use (p = 0.522). The comorbidity, reflected by the Charlson comorbidity index, showed higher values in multi-substance user compared to the control group consultation (p<0.001).

**Table 1:** Baseline characteristics.

	Total (n = 5,202)		Multi-substance user (n = 867)		Control (n = 4,335)		P-value
**Sociodemographic parameter**							
Age [year], median (IQR)	43.0	(34–49)	43.0	(34–49)	43.0	(34–49)	-
Sex [male], n (%)	3,108	(59.7)	580	(66.9)	2,528	(58.3)	<0.001
Private insurance, n (%)	385	(7.4)	2	(0.2)	383	(8.8)	<0.001
**Consultation acuity, n (%)**							
Triage							<0.001
Life-threatening	296	(5.7)	61	(7.0)	235	(5.4)	
High urgent	1,154	(22.2)	257	(29.6)	897	(20.7)	
Urgent	3,305	(63.5)	497	(57.3)	2,808	(64.8)	
Semi-urgent	329	(6.3)	44	(5.1)	285	(6.6)	
Non-urgent	118	(2.3)	8	(0.9)	110	(2.5)	
Resuscitation room	416	(8.0)	74	(8.5)	342	(7.9)	0.522
Walk-in	3,304	(63.5)	282	(32.5)	3,022	(69.7)	<0.001
**Consultation characteristics, n (%)**							
Night admission	1,760	(33.8)	289	(33.3)	1,471	(33.9)	0.733
Weekend admission	1,495	(28.7)	230	(26.5)	1,265	(29.2)	0.115
Season							0.234
Spring	1,199	(23.0)	191	(22.0)	1,008	(23.3)	
Summer	1,314	(25.3)	236	(27.2)	1,078	(24.9)	
Fall	1,303	(25.0)	227	(26.2)	1,076	(24.8)	
Winter	1,386	(26.6)	213	(24.6)	1,173	(27.1)	
Revisit	1,558	(30.0)	479	(55.2)	1,079	(24.9)	<0.001
Attending discipline							<0.001
Internal medicine	2,119	(40.7)	584	(67.4)	1,535	(35.4)	
Surgery	2,043	(39.3)	237	(27.3)	1,806	(41.7)	
Fast-Track^a^	588	(11.3)	40	(4.6)	548	(12.6)	
Ear-Nose-Throat	300	(5.8)	5	(0.6)	295	(6.8)	
Ophthalmology	152	(2.9)	1	(0.1)	151	(3.5)	
**Patient comorbidity, median (IQR)**							
Charlson comorbidity index [point]	0.0	(0–2)	0.0	(0–1)	0.0	(0–0)	<0.001

^a^ one physician treats low acuity, interdisciplinary ED visits

Recent intravenous drug use was documented in 32.0% of the multi-substance user consultations (n = 277) and in further 9.7% heroin consumption was documented, although the form of administration was not specified. In 58.4% of patients (n = 506), there was no explicit documentation of recent intravenous drug use or heroin use.

In total, 262 (30.2%) of the multi-substance user consultations were direct drug-related, 83 (9.6%) indirect and 522 (60.2%) were not drug-related.

The discharge diagnoses in the multi-substance users were broad and heterogeneous (as shown in S2 Table). The most common internal medicine diagnosis was Intoxication with somatic manifestation (15.2% of the multi-substance user consultations, n = 132) followed by Fever/SIRS/Sepsis (10.1%) and pneumonia (7.6%). The most common surgical diagnosis was trauma (8.3%, n = 72) and an additional psychiatric diagnosis was found in 125 consultations (14.4%).

### ED resource consumption

#### Comparison with age-matched control group

In univariable analysis, the median total ED resources were significantly higher (p<0.001) in multi-substance users (762 TP, IQR 459–1226) compared to the age-matched control group (462 TP, IQR 196–833) (Table 2). Furthermore, higher resource needs of multi-substance user consultations were found in all studied resource subgroups apart from MRT scans, which were less often performed in multi-substance user consultations (1.7% vs. 4.4%, p<0.001).

**Table 2:** Primary and secondary outcomes in multi-substance users and age-matched control group. Median (IQR) or absolute number (per cent) where appropriate.

	Total (n = 5,202)		Multi-substance user (n = 867)		Control (n = 4,335)		P-value
Total ED resources [TP]	511	(222–896)	762	(459–1,226)	462	(196–833)	<0.001
**Resource subgroups**							
Physician work [TP]	282	(109–442)	380	(211–540)	258	(98–418)	<0.001
Physician patient time [min]	45	(20–70)	60	(30–80)	40	(20–65)	<0.001
Physician admin time [min]	20	(5–40)	30	(10–50)	15	(5–35)	<0.001
Physician medical report time [min]	11	(0–11)	11	(11–20)	11	(0–11)	<0.001
Nurse work [TP]	35	(0–62)	35	(0–93)	35	(0–44)	<0.001
Laboratory resources [TP]	76	(0–175)	167	(62–331)	62	(0–154)	<0.001
Radiology resources [TP]	15	(0–239)	67	(0–312)	0	(0–199)	<0.001
X-ray performed [yes]	1,829	(35.2%)	357	(41.2%)	1,472	(34.0%)	<0.001
Sonography performed [yes]	752	(14.5%)	161	(18.6%)	591	(13.6%)	<0.001
CT scan performed [yes]	1,109	(21.3%)	216	(24.9%)	893	(20.6%)	0.005
MRT scan performed [yes]	205	(3.9%)	15	(1.7%)	190	(4.4%)	<0.001
**Administrative outcomes**							
Total ED costs [Swiss Francs]	890	(489–1,453)	1,235	(798–1,903)	822	(451–1,345)	<0.001
Length of ED stay [min]	201	(124–319)	272	(176–418)	191	(118–298)	<0.001
Length of hospital stay [days]	0.2	(0.1–1.7)	0.5	(0.2–6.5)	0.2	(0.1–0.8)	<0.001
**Clinical outcomes**							
ICU admission [yes]	331	(6.4%)	107	(12.3%)	224	(5.2%)	<0.001
In-hospital mortality [yes]	47	(0.9%)	16	(1.9%)	31	(0.7%)	0.001

**Abbreviations:** CT, Computer Tomography; ED, Emergency Department; GMR, Geometric Mean ratio; ICU, Intensive Care Unit; min, minutes; TP, Tax Points [medical currency]

The geometric mean of the total ED resource consumption was 1.2 (95% CI: 1.1–1.3, p = 0.001) times higher in multi-substance user ED consultations compared to the age-matched control group in multivariable analysis (Table 3 and S3 Table): in the studied resource subgroups, positive associations between multi-substance user consultation and i) physician work (GMR 1.1, 95% CI: 1.1–1.3, p = 0.034), ii) admin time (GMR 1.2, 95% CI: 1.1–1.3, p = 0.001), iii) laboratory resources (GMR 1.4, 95% CI: 1.2–1.7, p<0.001) as well as iv) radiology resources (GMR 1.4, 95% CI: 1.1–1.7, p = 0.002) were found in multivariable analysis. The odds for performing an MRT scan was 0.3 (95% 0.2–0.6, p<0.001) times lower in multi-substance user consultations compared to the control group consultations, while the odds of performed X-ray and sonography were 1.4 times higher.

**Table 3:** Linear respectively logistic regression of the association between being a multi-substance user and the outcomes. All effect sizes are adjusted for sociodemographic parameter (age, sex, private insurance), consultation acuity (triage, resuscitation room, walk-in), consultation characteristics (night admission, weekend admission, season, revisit, and attending discipline) as well as Charlson comorbidity index.

	Effect size	(95% CI)	p-value
**Primary Outcome**			
Total resources [TP], GMR	1.18	(1.1–1.3)	0.001
**Secondary Outcomes**			
**Resource subgroups**			
Physician work [TP], GMR	1.14	(1–1.3)	0.034
Physician patient time [min], GMR	1.06	(1–1.2)	0.295
Physician admin time [min], GMR	1.17	(1.1–1.3)	0.001
Physician medical report time [min], GMR	1.07	(1–1.2)	0.116
Nurse work [TP], GMR	0.87	(0.7–1)	0.055
Laboratory resources [TP], GMR	1.41	(1.2–1.7)	<0.001
Radiology resources [TP], GMR	1.40	(1.1–1.7)	0.002
X-ray performed [yes], OR	1.38	(1.2–1.6)	<0.001
Sonography performed [yes], OR	1.44	(1.2–1.8)	0.001
CT scan performed [yes], OR	1.09	(0.9–1.3)	0.393
MRT scan performed [yes], OR	0.36	(0.2–0.6)	<0.001
**Administrative outcomes**			
Total ED costs [Swiss Francs], GMR	1.10	(1–1.2)	0.001
Length of ED stay [min], GMR	1.22	(1.2–1.3)	<0.001
Length of hospital stay [days], GMR	1.27	(1.2–1.4)	<0.001
**Clinical outcomes**			
ICU admission [yes], OR	1.30	(1–1.7)	0.081
In-hospital mortality [yes], OR	1.40	(0.7–3)	0.378

**Abbreviation:** CI, Confidence Interval, CT, Computer Tomography; ED, Emergency Department; GMR, Geometric Mean ratio; ICU, Intensive Care Unit; min, minutes; OR, Odds Ratio; TP, Tax Points [medical currency]

#### Predicting ED resource consumption in multi-substance user

Focussing the analysis on the multi-substance user, three more variables were added to predict the total ED resource consumption: i) heavy ED user, ii) intravenous drug use, and drug-related consultation.

Table 4 respectively S4 Table show the results of a multivariable linear regression to predict ED resource consumption in multi-substance user with stepwise removal of variables with p>0.2 respectively without removal: indirect and non-drug related consultations had higher ED resource consumption compared to drug-related consultations. Furthermore, internal medicine visits compared to all other disciplines as well as consultations with treatment in the resuscitation room and non-urgent consultations needed more resources.

**Table 4:** Linear regression of the association between being a multi-substance user and the total ED resource consumption (n = 867). From all included predictors, i) sociodemographic parameter (age, sex, private insurance), ii) consultation acuity variables (triage, resuscitation room, walk-in), iii) consultation characteristics parameters (drug-related, heavy-ED user, intravenous drug-use, night admission, weekend admission, season, revisit, and attending discipline) as well as the Charlson comorbidity index, those with p>0.2 were stepwise removed.

Total ED resources [TP]	GMR	(95% CI)	p-value
**Consultation acuity**			
Triage			
Life-threatening	1.00	(0.7–1.4)	0.985
High urgent	1.22	(1–1.4)	0.012
Urgent	1.00	base	
Semi-urgent	0.71	(0.5–1)	0.029
Non-urgent	3.89	(1.8–8.4)	0.001
Resuscitation room [yes]	1.57	(1.2–2.1)	0.001
**Consultation characteristics**			
Drug related			
Direct	1.00	base	
Indirect	1.59	(1.2–2)	<0.001
Not-related	1.19	(1–1.4)	0.019
Discipline			
Internal medicine	1.00	base	
Surgery	0.83	(0.7–1)	0.014
Fast-Track	0.17	(0.1–0.2)	<0.001
Ear-Nose-Throat	0.13	(0.1–0.3)	<0.001
Ophthalmology	0.11	(0–0.8)	0.028

Abbreviation: CI, Confidence Interval; ED, Emergency Department; GMR, Geometric Mean ratio; TP, Tax Points [medical currency]

### Administrative and clinical outcomes

In univariable analysis the three studied administrative outcomes (total ED costs, length of ED stay, and length of hospital stay) as well as the two studied clinical outcomes (ICU admission and in-hospital mortality) were significantly increased (p≤0.001) in multi-substance user consultations (see Table 2). After adjustment of this association for the potential confounder, a significant positive association was found in regard to length of ED stay and length of hospital stay as well as total ED costs (Table 3). ICU admission (OR 1.3, 95%, 1.0–1.7, p = 0.081) and in-hospital mortality (OR 1.4, 95% CI: 0.7–3.0, p = 0.378) did not differ significantly between the study groups.

### Sensitivity analysis

A matched analysis using a conditional multivariable linear regression analysis did only very slightly change the effect size between total ED resources and multi-substance user consultation presented in Table 3 (GMR: 1.2, 95% CI: 1.1–1.3, p = 0.001).

Restricting the main analysis (Tables 3 and 4) to the first consultation of an included multi-substance user to take into account that follow-up visits of a multi-substance user might not be independent events, did only slightly change the found results (S5 and S6 Table).

## Discussion

Compared to a control group in confounder adjusted regression analysis, ED consultations of multi-substance users generated higher overall costs and needed more overall ED resources, especially high levels of physician and laboratory resources. Despite the high resource consumption, multi-substance users had more revisits, longer ED and in-hospital stays but no increased mortality or ICU admissions when controlled for confounders.

ED visits of multi-substance users consumed significantly more resources than non-multi-substance users. Our detailed analyses showed that this is mainly due to use of physicians’ time and laboratory resources. This may be even more pronounced if one considers the lower use of expensive procedures such as MRT in this subgroup.

Increased morbidity associated with substance use, particularly the increased risk of immune-deprived status caused by chronic infectious diseases (hepatitis, HIV, tuberculosis) [18], is likely to account for the greater clinical severity of these cases, also demonstrated with the higher Charlson comorbidity index in our study, as well as for unusually complicated or rare disease entities. These more severe and complex or rare cases may contribute to the increased physician and laboratory resources needed in this population. Interestingly, the indirectly drug related visits (e.g. intoxicated trauma, alcoholic pancreatitis) turned out to be an even more relevant cost factor compared to directly drug related visits (e.g. acute intoxications). This is part of the known high burden of comorbidities in this patient group, as confirmed in our study. Thus, the improvement of the treatment of the secondary drug effects should be focused on and not mainly the acute intoxications, although the acute intoxications might be often in focus of the media or ED providers.

In addition to general increased morbidity, psychiatric comorbidity may be one of the reasons for excessive physicians’ work and time consumption especially in multi-substance users with frequent ED visits [7,8,19]. Detection of psychiatric comorbidity can therefore be crucial to identify patients at increased risk of re-admission [20].

This increased number of psychiatric comorbidities might come together with the known difficult treatment of patients with disruptive behaviour, as is often experienced within the care of multi-substance users or patients with additional mental health disorders. Patients with disruptive behaviour are known to be difficult to diagnose and are in danger of receiving incorrect or delayed diagnoses [21,22]. Sensitization of ED physicians about this problem as well as identification of individuals at risk may prevent errors and reduce unnecessary resource use. Another approach to reduce physicians’ work and time consumption in “hard-to-handle” patients may be early interdisciplinary teamwork. Further research is needed to better understand this problem in the patient group of multi-substance users and develop strategies to overcome this bias.

Although the ED work up for the group of multi-substance users devours more resources and is more expensive compared to the matched control group, the multi-substance users had more revisits compared to the control group. This may be attributed to the significantly higher morbidity which is shown in the higher Charlson comorbidity index in our study and in line with the literature [13,14]. Another explanation may be the lower socioeconomic status [23], mirrored in the lower number of privately insured patients in our studied multi-substance user population or a result of a suboptimal ambulatory care of this specific patient group.

Previous studies have demonstrated that substance use is associated with more frequent ED consultations [3,8,24]. Multi-substance users are thought to use medical services in EDs particularly because of its 24/7 availability around the clock. Some authors have claimed that one explanation for this might be that multi-substance users present to the ED comparatively more frequently at night [25]. This could not be replicated in our analyses.

It has been shown that multi-substance users misuse the ED as a substitute for primary care [6]. Socioeconomic factors, such as homelessness, social isolation, being single, and social welfare dependency are associated with more frequent and heavy ED use [7,9], as well as chronic diseases [9]. For these patients, the major obstacle is probably not the unavailability of primary care but problems of access.

Consequently, ED interventions which identify patients at risk and target those factors might help to facilitate access to primary care and decrease the number of frequent emergency consultations. Reviews about interventions and screening for substance use disorders showed a reduction in alcohol consume even months after a very limited short time intervention at the ED [20,26]. Important steps to improve the identification of those patients and improve their ED treatment can be the specialisation of physicians [27] and, even more important, education in order to raise awareness of all ED physicians.

Closer collaboration with primary and secondary care services–in particular those also specialised in treatment of comorbid psychiatric and substance use disorders–may be mandatory. While transitional care clinics have given promising results and closer linkage between ED and primary care services may facilitate follow-up and reduce multiple ED presentations [28], substance-use disorder patients may be a particularly challenging subgroup of patients. Thus, investing more resources for such interventions up front may reduce resource consumption through revisits. How much any such intervention would cost depends on local factors such as case load, availability of a social worker, etc. We would however argue that whatever the specific costs are, these are likely to be compensated if they prevent just one out of 4 revisits, as any such intervention would–in our setting–not exceed the price of a fourth of an average visit.

Future research should evaluate the cost effectiveness of any potential intervention and focus on interventional strategies for the early detection of “at-risk” patients and subsequent interdisciplinary and target-oriented management of these individuals, as well as intensified collaboration between EDs and primary care services.

### Limitations

The limitations of our study include the missing or unknown real figure for the specific substances the patients were consuming. As previous studies have shown, associated risk of increased morbidity and mortality differs widely between specific substances [13,14]. We only matched for age and adjusted the remaining baseline difference between cases and controls, with a multivariable analysis. This allowed to study the impact of the potential confounder on the outcome.

Another limitation is the wide variation in the definition of multi-substance user in the research and official use. In our study, we focused on patients with abuse of multi-substances mainly alcohol, illicit drugs, and opioid/benzodiazepine abuse. This is not in line with the definition of the Swiss Federal Office of Public Health, where simultaneous daily tobacco and occasional alcohol use itself already is classified as multi-substance use. Thus, the characteristics of consultations by multi-substance users could not be set into the context of the background population to further identify multi-substance users at risk for ED consultations. Furthermore, we used a key-word search in the first step to identify cases, which might have led to bias.

As this was a single centre study in a university hospital, transferability to other settings may be limited and further research is warranted here. As diagnoses were extracted retrospectively from medical reports, there is a risk of missed cases due to incomplete detection or reporting of diagnoses. However, routine medical reports of our university hospital ED must contain detailed documentation, including a comprehensive medical history that covers all previous primary care and the available inpatient medical reports.

Additional strengths of our study include the high number of included cases as well as the reasonable observation period of three years, and this suggests that our findings may be more widely applicable.

## Conclusions

Compared to an age-matched control group, ED consultations of multi-substance users were more expensive and resource intensive, especially the indirectly drug related visits, particularly with respect to physician time adjusted for important confounding variables.

Multi-substance users visited the ED more often and stayed longer at the ED and in-hospital. The findings of our study underline the importance of the patient group of multi-substance users. Additional efforts should be made to improve the ED care and follow-up care of those patients. Special interventions should target this patient in order to decrease the high frequency and costs of emergency consultations of multi-substance users.

### Ethical considerations

The study was approved by the regional ethics committee of the Canton of Bern, Switzerland (KEK-BE-2018-00359 and 2018–00198). The data was anonymized prior to analysis. The ethics committee waived the requirement for informed consent.

## Supporting information

S1 File Dataset of the study. (XLSX)

S1 Table Diagnostic groups. Table showing the diagnostic groups used to classify the different disease patterns.
(PDF)

S2 Table Established diagnosis groups. Table showing the established diagnoses of multi-substance users.
(PDF)

S3 Table Multivariable linear regression of the association between being a multi-substance user and the primary outcome: total ED resources adjusted for the potential confounder. (PDF)

S4 Table Linear regression of the association between being a multi-substance user and the total ED resource consumption (n = 867). (PDF)

S5 Table Linear respectively logistic regression of the association between being a multi-substance user and the outcomes restricted to the first consultation of a multi-substance user. (PDF)

S6 Table Linear regression of the association between being a multi-substance user and the total ED resource consumption (n = 460). (PDF)
