# Supplementary material for: Resource consumption of multi-substance users in the emergency room: A neglected patient group
Source: PLoS One. 2019 Sep 26;14(9):e0223118. doi: 10.1371/journal.pone.0223118 (PMC6763017; doi:10.1371/journal.pone.0223118)
Supplement: S1 Table — Table showing the diagnostic groups used to classify the different disease patterns. (PDF) [file pone.0223118.s002.pdf]

Supplement 1. Diagnostic groups

**Surgical**	**Medical**	**Psychiatric**
Trauma (fall or any trauma leading to cut/stab wound or fracture)<br>Laceration, cut, stab wound<br>Fracture<br>Chemical burn/corrosive injury burn<br>Cutaneous ulceration<br>Abscess<br>Syringe abscess<br>Intracranial haemorrhage<br>Urological diagnosis<br>Other musculoskeletal diagnosis<br>Other	Erysipelas, cutaneous infection, thrombophlebitis<br>Endocarditis<br>Pneumonia<br>Respiratory tract infection (viral), pleuritis, dyspnoea<br>Pancreatitis, hepatological dx<br>DVT/PE<br>COPD<br>Epileptic seizure<br>Withdrawal<br>Gastrointestinal haemorrhage<br>Abdominal dx, excl. haemorrhage<br>Intoxication with somatic manifestation, cardiovascular dx<br>Fever, SIRS, sepsis<br>Other	Suicidal tendency<br>Hetero-aggression<br>Self-endangerment<br>Underlying psychiatric dx<br>Intoxication without somatic manifestation<br>Other

Abbreviations: COPD, chronic obstructive pulmonary disease; DVT, deep venous thrombosis; dx, diagnosis; excl., excluded; PE, pulmonary embolism, SIRS, systemic inflammatory response syndrome.
