# Supplementary material for: Resource consumption of multi-substance users in the emergency room: A neglected patient group
Source: PLoS One. 2019 Sep 26;14(9):e0223118. doi: 10.1371/journal.pone.0223118 (PMC6763017; doi:10.1371/journal.pone.0223118)
Supplement: S2 Table — Table showing the established diagnoses of multi-substance users. (PDF) [file pone.0223118.s003.pdf]

Supplement 2. Established diagnosis groups

	Total, n (%)	
**Internal medicine diagnosis groups**		
Intoxication with somatic manifestation	132	(16.7)
Fever, SIRS, sepsis	88	(11.1)
Pneumonia	66	(8.4)
Erysipelas, cutaneous infection, thrombophlebitis	60	(7.6)
Pancreatitis, hepatological diagnosis	51	(6.5)
Abdominal diagnosis, excl. Haemorrhage	49	(6.2)
Respiratory tract infection (viral) including pleuritis, dyspnoea	34	(4.3)
Withdrawal	30	(3.8)
Epileptic seizure	26	(3.3)
DVT/PE	26	(3.3)
Cardiovascular diagnosis	23	(2.9)
Endocarditis	9	(1.1)
COPD	9	(1.1)
Gastrointestinal haemorrhage	7	(0.9)
Ischemic stroke	2	(0.3)
Other	178	(22.5)
**Total**	790	(100.0)
**Surgical diagnosis groups**		
Trauma (fall or any trauma leading to cut/stab wound or fracture)	72	(19.6)
Fracture	54	(14.7)
Other musculoskeletal diagnosis	44	(12.0)
Laceration, cut, stab wound	39	(10.6)

Syringe abscess	33	(9.0)
Cutaneous ulceration	25	(6.8)
Abscess	25	(6.8)
Intracranial haemorrhage	8	(2.2)
Urological diagnosis	7	(1.9)
Chemical burn/corrosive injury, burn	3	(0.8)
Other	57	(15.5)
**Total**	367	(100.0)

**Psychiatric diagnosis groups**		
Underlying psychiatric diagnosis	32	(25.6)
Intoxication without somatic diagnosis	28	(22.4)
Suicidal tendency	19	(15.2)
Hetero-aggression	13	(10.4)
Self-endangerment	9	(7.2)
Other	24	(19.2)
**Total**	125	(100.0)

Abbreviations: COPD, chronic obstructive pulmonary disease; DVT, deep venous thrombosis; dx, diagnosis; excl., excluded; PE, pulmonary embolism, SIRS, systemic inflammatory response syndrome.
