# Supplementary material for: Resource consumption of multi-substance users in the emergency room: A neglected patient group
Source: PLoS One. 2019 Sep 26;14(9):e0223118. doi: 10.1371/journal.pone.0223118 (PMC6763017; doi:10.1371/journal.pone.0223118)
Supplement: S3 Table — (PDF) [file pone.0223118.s004.pdf]

Supplement 3. Multivariable linear regression of the association between being a multi-substance user and the primary outcome: total ED resources adjusted for the potential confounder and removing variables with p>0.2.

Total ED resources [TP]	GMR	(95% CI)	p-value
**Multi-substance user consultation**	1.18	(1.1 - 1.3)	0.001
**Sociodemographic parameter**			
Age [year]	1.00	(1 - 1)	0.192
**Consultation acuity**			
Triage			
Life-threatening	1.50	(1.3 - 1.8)	<0.001
High urgent	1.42	(1.3 - 1.5)	<0.001
Urgent	1.00	base	
Semi-urgent	0.48	(0.4 - 0.6)	<0.001
Non-urgent	0.93	(0.7 - 1.2)	0.512
Resuscitation room [yes]	1.84	(1.6 - 2.1)	<0.001
Walk-in [yes]	0.84	(0.8 - 0.9)	<0.001
**Consultation characteristics**			
Discipline			
Internal medicine	1.00	base	
Surgery	0.76	(0.7 - 0.8)	<0.001
Fast-Track	0.43	(0.4 - 0.5)	<0.001
Ear-Nose-Throat	0.16	(0.1 - 0.2)	<0.001
Ophthalmology	0.12	(0.1 - 0.1)	<0.001
**Patient characteristics**			
Charlson comorbidity index [point]	1.05	(1 - 1.1)	0.005

Abbreviation: CI, Confidence Interval; ED, Emergency Department; GMR, Geometric Mean ratio; TP, Tax Points [medical currency]
