# Supplementary material for: Resource consumption of multi-substance users in the emergency room: A neglected patient group
Source: PLoS One. 2019 Sep 26;14(9):e0223118. doi: 10.1371/journal.pone.0223118 (PMC6763017; doi:10.1371/journal.pone.0223118)
Supplement: S4 Table — (PDF) [file pone.0223118.s005.pdf]

Suuplement 4. Linear regression of the association between being a multi-substance user and the total ED resource consumption (n=867).

Total ED resources [TP]	GMR	(95% CI)	p-value
**Multi-substance user consultation characteristic**			
Intravenous drug use	1.05	(0.9 - 1.2)	0.344
Heavy ED user	0.94	(0.8 - 1.1)	0.476
Drug related			
Direct	1.00	base	
Indirect	1.54	(1.2 - 2)	0.001
Not-related	1.18	(1 - 1.4)	0.032
**Sociodemographic parameter**			
Age [year]	1.00	(1 - 1)	0.436
Sex [male]	1.02	(0.9 - 1.2)	0.827
Private incurance [yes]	2.24	(0.6 - 8.9)	0.251
**Consultation acuity**			
Triage			
Life-threatening	0.99	(0.7 - 1.4)	0.971
High urgent	1.19	(1 - 1.4)	0.028
Urgent	1.00	base	
Semi-urgent	0.70	(0.5 - 1)	0.025
Non-urgent	3.63	(1.7 - 7.9)	0.001
Resuscitation room [yes]	1.54	(1.2 - 2)	0.002
Walk-in [yes]	0.90	(0.8 - 1)	0.161
**Consultation characteristics**			
Night admission [yes]	0.94	(0.8 - 1.1)	0.369
Weekend admission [yes]	0.99	(0.9 - 1.2)	0.916
Season [base: spring]			
Spring	1.00	base	
Sommer	1.01	(0.8 - 1.2)	0.909
Fall	0.98	(0.8 - 1.2)	0.837

Winter	1.12	(0.9 - 1.4)	0.258
Revisit [yes]	1.11	(1 - 1.3)	0.158
Discipline			
Internal medicine	1.00	base	
Surgery	0.83	(0.7 - 1)	0.019
Fast-Track	0.18	(0.1 - 0.3)	<0.001
Ear-Nose-Throat	0.14	(0.1 - 0.3)	<0.001
Ophthalmology	0.12	(0 - 0.8)	0.031
**Patient characteristics**			
Charlson comorbidity index [point]	1.00	(1 - 1.1)	0.906

Abbreviation: CI, Confidence Interval; ED, Emergency Department; GMR, Geometric Mean ratio; TP, Tax Points [medical currency]
