# Supplementary material for: Resource consumption of multi-substance users in the emergency room: A neglected patient group
Source: PLoS One. 2019 Sep 26;14(9):e0223118. doi: 10.1371/journal.pone.0223118 (PMC6763017; doi:10.1371/journal.pone.0223118)
Supplement: S5 Table — (PDF) [file pone.0223118.s006.pdf]

Supplement 5. Linear respectively logistic regression of the association between being a multi-substance user and the outcomes restricted to the first consultation of a multi-substance user (n=458).

All ratios are adjusted for sociodemographic parameter (age, sex, private insurance), consultation acuity (triage, resuscitation room, walk-in), consultation characteristics (night admission, weekend admission, season, and discipline) as well as charlson comorbidity index.

	Effect size	(95% CI)	p-value
**Primary Outcome**			
Total resources [TP], GMR	1.16	(1 - 1.3)	0.026
**Secondary Outcomes**			
**Resource subgroups**			
Physician work [TP], GMR	1.14	(1 - 1.3)	0.103
Physician patient time [min], GMR	1.02	(0.9 - 1.2)	0.728
Physician admin time [min], GMR	1.14	(1 - 1.3)	0.046
Physician medical report time [min], GMR	1.06	(0.9 - 1.2)	0.285
Nurse work [TP], GMR	0.75	(0.6 - 0.9)	0.004
Laboratory resources [TP], GMR	1.39	(1.1 - 1.7)	0.003
Radiology resources [TP], GMR	1.30	(1 - 1.7)	0.078
X-ray performed [yes], OR	1.29	(1 - 1.6)	0.033
Sonography performed [yes], OR	1.39	(1 - 1.9)	0.028
CT scan performed [yes], OR	1.03	(0.8 - 1.3)	0.819
MRT scan performed [yes], OR	0.31	(0.1 - 0.7)	0.005
**Administrative outcomes**			
Total ED costs [Swiss Francs], GMR	1.07	(1 - 1.2)	0.079
Length of ED stay [min], GMR	1.21	(1.1 - 1.3)	<0.001
Length of hospital stay [days], GMR	1.23	(1.1 - 1.3)	<0.001
**Clinical outcomes**			
ICU admission [yes], OR	1.23	(0.8 - 1.8)	0.303
In-hospital mortality [yes], OR	1.16	(0.5 - 2.8)	0.737

Abbreviation: CI, Confidence Interval, CT, Computer Tomography; ED, Emergency Department; GMR, Geometric Mean ratio; ICU, Intensive Care Unit; min, minutes; OR, Odds Ratio; TP, Tax Points [medical currency]
