# Supplementary material for: Resource consumption of multi-substance users in the emergency room: A neglected patient group
Source: PLoS One. 2019 Sep 26;14(9):e0223118. doi: 10.1371/journal.pone.0223118 (PMC6763017; doi:10.1371/journal.pone.0223118)
Supplement: S6 Table — (PDF) [file pone.0223118.s007.pdf]

Supplement 6. Linear regression of the association between being a multi-substance user and the total ED resource consumption (n=458).

From all included predictors, i) sociodemographic parameter (age, sex, private insurance), ii) consultation acuity variables (triage, resuscitation room, walk-in), iii) consultation characteristics parameters (drug-related, heavy-ED user, intravenous drug-use, night admission, weekend admission, season, revisit, and attending discipline) as well as the Charlson comorbidity index, those with p>0.2 were stepwise removed.

Total ED resources [TP]	GMR	(95% CI)	p-value
**Sociodemographic parameter**			
Age [year]	1.01	(1 - 1)	0.082
Private insurance	2.65	(0.6 - 11.2)	0.184
**Consultation acuity**			
Triage			
Life-threatening	0.99	(0.7 - 1.5)	0.957
High urgent	1.21	(1 - 1.5)	0.085
Urgent	1.00	base	
Semi-urgent	0.83	(0.5 - 1.3)	0.383
Non-urgent	3.51	(1 - 12.8)	0.058
Resuscitation room [yes]	1.59	(1.1 - 2.4)	0.023
**Consultation characteristics**			
Heavy ED user	0.77	(0.5 - 1.1)	0.148
Drug related			
Direct	1.00	base	
Indirect	1.49	(1 - 2.1)	0.032
Not-related	1.23	(1 - 1.5)	0.071
Discipline			
Internal medicine	1.00	base	
Surgery	0.73	(0.6 - 0.9)	0.006
Fast-Track	0.20	(0.1 - 0.4)	<0.001
Ear-Nose-Throat	0.15	(0.1 - 0.4)	<0.001
Ophthalmology	0.11	(0 - 0.8)	0.033

Abbreviation: CI, Confidence Interval; ED, Emergency Department; GMR, Geometric Mean ratio; TP, Tax Points [medical currency]

ln_total_res	GMR	Std. Err.	t	P>t	[95% Conf.	Interval]
age	1.008374	0.0048233	1.74	0.082	0.9989393	1.0178
private	2.650233	1.939389	1.33	0.184	0.629044	11.1657
triage						
Life-threateni	0.9884886	0.2102856	-0.05	0.957	0.6507206	1.501581
High urgent	1.213037	0.1355489	1.73	0.085	0.9738602	1.510954
Urgent	1	(base)				
Semi-urgent	0.8271648	0.1796057	-0.87	0.383	0.5398334	1.267431
Non-urgent	3.505533	2.310205	1.9	0.058	0.9599702	12.80119
Resus	1.593505	0.3247065	2.29	0.023	1.06765	2.378362
heavy_users	0.7694832	0.1392803	-1.45	0.148	0.5391436	1.098232
drug_related_1direct_2indir_3no						
Direct	1	(base)				
Indirect	1.490613	0.2758189	2.16	0.032	1.036164	2.144378
No	1.226813	0.1383717	1.81	0.071	0.9828972	1.531259
discipline						
Internal me	1	(base)				
Surgery	0.7342476	0.0824904	-2.75	0.006	0.5887761	0.9156614
Fast-Track	0.197452	0.058307	-5.49	0	0.110513	0.352783
Ear-Nose-T	0.1532026	0.0792753	-3.63	0	0.0554114	0.4235779
Ophthalmolc	0.1107799	0.1140257	-2.14	0.033	0.0146526	0.8375445
